# Do research findings on schema-based instruction translate to the classroom?

**DOI:** 10.1007/s40037-015-0225-5

**Published:** 2015-10-26

**Authors:** Sarah Blissett, Mark Goldszmidt, Matt Sibbald

**Affiliations:** 1 HGH- McMaster Clinic, Hamilton General Hospital, Hamilton, Ontario Canada; 2 Department of Medicine, Western University, London, Ontario Canada

**Keywords:** Cognitive load theory, Schemas, Translational research

## Abstract

**Introduction:**

Schema-based instruction has been shown to improve diagnostic performance and reduce cognitive load. However, to date, this has only been studied in controlled research settings. More distractions in classrooms may limit generalizability to real-world settings. We evaluated whether schema-based instruction would maintain its effects on cognitive load optimization and performance in a classroom.

**Methods:**

Focused on the approach of interpreting cardiac auscultation findings, 101 first-year medical students at Western University were randomized to receive a traditional (*n* = 48) or a schema-based lecture (*n* = 53). Students completed four written questions to test diagnostic performance and a cognitive load assessment at the end of the lecture. Diagnostic performance and cognitive load were compared with independent t-tests.

**Results:**

Schema-based instruction was associated with increased diagnostic performance on written questions (64 ± 22 % vs 44 ± 25 % *p* < 0.001) and reduced intrinsic cognitive load (mean difference = 15 %, standard error 3 %, *p* < 0.001). There was no significant difference in reported extraneous (*p* = 0.36) or germane (*p* = 0.42) cognitive load.

**Conclusions:**

Our results demonstrate that schema-based instruction can be used to reduce intrinsic load and improve diagnostic performance in a real-world classroom setting. The results would be strengthened by replication across other locations and topics.

## Introduction

Applying principles from cognitive psychology to instructional design in medical education has demonstrated improvements in performance [1]. Within research studies, applications of cognitive psychology have been associated with increased diagnostic performance [2–6]. However, studies exploring the translation of beneficial interventions to the classroom are limited, despite criticism over the generalizability of research results to practical teaching settings.

**Fig. 1:**
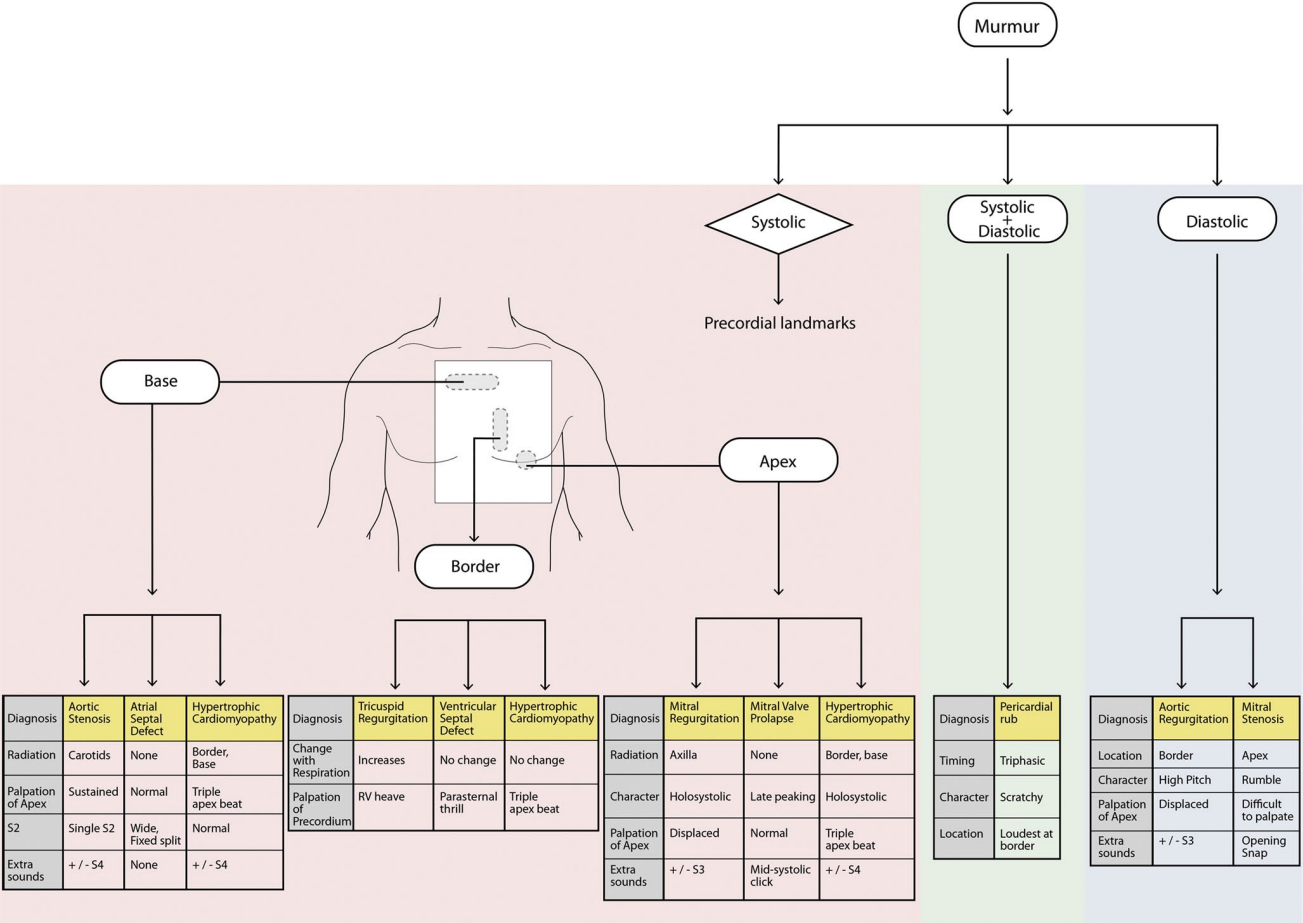
Schema used in schema-based lecture

In medicine, schemas are described in relationship to diagnoses. In this context, a schema is a diagnostic algorithm that organizes a differential diagnosis by the presenting complaint and provides clinically relevant discriminating features to distinguish similar diagnoses (Fig. 1; [5]). In contrast, traditional instructional formats organize information by diagnosis, often in lists of unlinked facts.

Patients typically present with an undifferentiated clinical problem rather than a known diagnosis. Information provided in a schema is more conducive to assessing undifferentiated complaints as it is organized according to the presenting complaint; however, the information in a traditional format must be reorganized. Providing information in traditional formats emphasizes comprehensiveness, whereas organization into a schema focuses the learner on the features to distinguish diagnoses. Presenting a differential diagnosis in a schema encourages comparing and contrasting between diagnoses, mirroring the decision-making process required when assessing patients.

The improved diagnostic accuracy associated with schema use in research settings is well described [2–6]. Improvements of up to 35 % in diagnostic accuracy on practical tasks and 20 % on written questions have been observed with schemas [2].

Cognitive load theory (CLT) can be used to explain the improved diagnostic performance seen with schemas. CLT is based on the premise that working memory is limited [7]. Cognitive load can be optimized by addressing each of its subcomponents: intrinsic, extraneous and germane cognitive load. Intrinsic load describes the inherent difficulty of the task. Schemas optimize intrinsic load by reducing the total number of facts, grouping similar information and organizing information in a clinically relevant format. Extraneous cognitive load refers to any mental effort that does not contribute to learning. It is ideally minimized. Schemas may decrease extraneous load by organizing the information around the presenting complaint, avoiding the reorganization of information required if provided by individual diagnoses. Germane cognitive load describes the processing required to transfer information from working memory to storage in long-term memory. Schemas optimize germane cognitive load by chunking information and associating similar diagnoses. Schema-based instruction has been associated with lower overall cognitive load as compared with traditional methods of instruction [6].

Prior studies have evaluated interventions under research protocols with volunteer subjects. The controlled designs may limit generalizability of the results to a classroom setting. There may be more distractions in a classroom, such as internet resources, classmates or mobile technology [8]. As such, classrooms may have higher extraneous load than research conditions.

There is limited evidence supporting the application of CLT to the classroom. Issa et al. [9] studied the translation of multimedia design principles into clerkship lectures, finding improved written performance when students were instructed with lectures that optimized multimedia design and cognitive load. In another small study, lectures modified to optimize cognitive load were associated with higher knowledge scores and lower cognitive load as compared with traditional lectures [10]. In an observational study, participants who used a schema for metabolic disturbances when answering written questions had higher diagnostic accuracy scores. [3].

It is unclear how the increased extraneous cognitive load of a classroom environment impacts beneficial effects of schema-based instruction. The potential interactions between intrinsic, extraneous and germane load have been debated [11, 12]. The increased extraneous load in the classroom could consume working memory and negate any potential reductions in intrinsic load [12]. Alternatively, the benefits of schemas may be accentuated with increased extraneous load because the optimized intrinsic load requires less working memory. Distractions could have a greater impact on traditional methods of teaching that require more working memory.

The purpose of this study was to evaluate whether schema-based instruction would maintain its effects on cognitive load and diagnostic performance in a classroom setting. We hypothesized that the schema-based lecture would decrease intrinsic load and increase diagnostic performance.

## Methods

### Participants and procedure

The study was embedded within a lecture on structural heart disease in the Cardiology curriculum for first-year medical students at Western University. The participants had competed 5 months of medical school on topics unrelated to Cardiology prior to the study.

We utilized a single-blind randomized controlled trial design. Students were randomized using a random number generator.

Both lectures were designed around the same objectives: describe and classify normal heart sounds, abnormal heart sounds and cardiac murmurs. Ten structural lesions (aortic stenosis, atrial septal defect, hypertrophic cardiomyopathy, tricuspid regurgitation, ventricular septal defect, mitral regurgitation, mitral valve prolapse, aortic regurgitation, mitral stenosis and pericardial rub) were described.

The lectures contained the same number of slides (81 slides) with the same average number of words per slide (21 words). Both lectures were given in a standard 1 hour lecture time frame in lecture halls used in the undergraduate medical curriculum at Western University. The lectures were given simultaneously by two different lecturers.

Both lectures began with a review of the physiology of normal heart sounds, followed by a discussion of abnormal heart sounds.

To ensure that no group was significantly disadvantaged prior to the course examination, both lectures were repeated immediately following data collection.

### Control lecture

The details of each lesion were listed in succession. The lecturer outlined the character, radiation, extra sounds, and palpable findings for each murmur.

A junior faculty member who had provided this lecture in previous years delivered the lecture.

### Experimental lecture

The schema-based lecture presented the same introduction. Murmurs were presented according to timing and location using an adaptation of a published schema (Fig. 1; [2]). For example, when systolic murmurs at the base of the heart were described, a differential diagnosis of aortic stenosis, atrial septal defect and hypertrophic cardiomyopathy was presented. The lecturer then explained the pathophysiology and associated findings that would allow differentiation between the differential provided.

The lecture was given by a junior lecturer who had used this schema in previous research studies [2].

### Instruments

Immediately following the lecture, participants completed a cognitive load assessment about the lecture and a four-item test assessing diagnostic performance.

The cognitive load assessment was based on a previously validated tool [13] to assess the subcomponents of cognitive load. Each subcomponent consisted of 3–4 individual questions on a 10-point scale. Subcomponent scores were added to arrive at a score for intrinsic (maximum 30 points), extraneous (maximum 30 points) and germane (maximum 40 points) cognitive load.

The four written multiple-choice questions were generated by three experts in cardiac auscultation. The questions provided details of the cardiac physical exam. Participants provided the diagnosis without the aid of any instructional materials.

Three weeks following the study date, participants rated the lecturers on a 7-point scale (1 = below expectations, 4 = average, 7 = outstanding).

### Statistics

Independent t-tests were performed to compare the cognitive load assessments and written questions. Lecturer ratings were compared with a paired t-test.

Ethics approval was granted by the Western University Research Ethics Board.

## Results

Of 133 students, 101 participated (76 %), with 48 randomized to the traditional lecture and 53 randomized to the schema-based lecture.

Schema-based instruction was associated with increased performance on the diagnostic performance test (64 % ± 22 vs 44 % ± 25, *p* < 0.001).

Participants rated intrinsic cognitive load lower with the schema-based lecture (20 vs 24 points, mean difference = 15 %, standard error 3 %, *p* < 0.001). There was no significant difference in reported extraneous (11 vs. 12 points, mean difference = 3 %, standard error = 3 %, *p* = 0.36) or germane (22 vs 21 points, mean difference = 3 %, standard error = 4.5 %, *p* = 0.42) load.

Fifty-nine (59 %) participants evaluated the lecturers. Participants rated both lecturers similarly (traditional lecture: 4.98 vs schema-based lecture: 5.25, *p* = 0.114).

## Discussion

Mirroring findings from research-oriented settings, we identified an increase in diagnostic performance and a reduction in intrinsic cognitive load with schema-based instruction in a classroom setting.

We observed an increase in diagnostic performance of 20 % in the schema group. This effect size is comparable to a small study of lectures optimized using cognitive load theory (15 %) [10]. This effect size is also in keeping with a study that identified an increase in written diagnostic accuracy of 20 % with the use of a similar schema for murmur identification conducted in research settings [2]. The similarity of effect sizes suggests that the potential increased extraneous load of the classroom setting did not undermine the optimized intrinsic load of the schemas.

The reduction in intrinsic load may reflect the different organizational approaches for the traditional and schema-based lectures. The schema-based lecture focused on discriminating facts and contextualized pathophysiological explanations for abnormal cardiac findings into discussion of differential diagnosis, whereas the traditional lecture focused on describing the conditions. While the reduction in intrinsic load could also be attributed to lecturer attributes, the similarities between the two lecturer ratings argue that the organizational approach likely had more impact on the intrinsic load measurements.

The similar extraneous load measurements support the argument that performance differences were due to the organizational approach to the material rather than differences in the lecturer’s style or different amounts of classroom distractions.

There are several limitations to this study. Firstly, it was conducted at a single centre and results would be strengthened by replication at other sites. Secondly, two different lecturers provided the lectures and differences in style could have influenced the results. We attempted to control the effect of lecturer style by controlling slide content. Thirdly, the outcome measure for diagnostic performance was limited to four questions and not standardized; however no standardized questions on cardiac auscultation exist in the literature. Lastly, delayed performance was not measured. Providing the complementary lecture following the study lecture limited our ability to conduct delayed testing.

## Conclusion

In conclusion, schema-based instruction maintained its beneficial effects in a classroom environment. Our findings support the translation of applied educational theory to the classroom.
